# Association between the quality of inner cell mass and first trimester miscarriage after single blastocyst transfer

**DOI:** 10.1186/s12958-020-00595-y

**Published:** 2020-05-12

**Authors:** Dayuan Shi, Jiawei Xu, Meixiang Zhang, Wenbin Niu, Hao Shi, Guidong Yao, Ying Li, Nan Zhang, Yingpu Sun

**Affiliations:** 1 Center for Reproductive Medicine, The First Affiliated Hospital of Zhengzhou University, Zhengzhou, China; 2 Henan Key Laboratory of Reproduction and Genetics, The First Affiliated Hospital of Zhengzhou University, Zhengzhou, China; 3 Henan Provincial Obstetrical and Gynecological Diseases (Reproductive Medicine) Clinical Research Center, The First Affiliated Hospital of Zhengzhou University, Zhengzhou, China; 4 Henan Engineering Laboratory of Preimplantation Genetic Diagnosis and Screening, The First Affiliated Hospital of Zhengzhou University, Zhengzhou, China; 5 The Academy of Medical Science, Zhengzhou University, Zhengzhou, China

**Keywords:** Blastocyst morphology, Miscarriage, Chromosome karyotype, Single blastocyst transfer

## Abstract

**Background:**

The blastocyst morphology provided valuable roles for predicting pregnancy and live birth, but was still not fully understood for evaluating miscarriage. The aim of this study was to explore the association between blastocyst morphologic evaluation and first trimester miscarriage combined with karyotype of miscarried conceptus.

**Methods:**

This retrospective cohort study included a total of 2873 clinical pregnancy cycles with single blastocyst transfer performed from January 2013 to April 2019. Chromosome karyotype of miscarried conceptus was analyzed via single nucleotide polymorphism array analysis. Miscarriage and karyotype of miscarried conceptus associated with blastocyst morphology were analyzed by chi-square and logistic regression analysis.

**Results:**

A total of 354 (12.3%) cycles resulted in first trimester miscarriage. Miscarriage rates increased with trophectoderm (TE) grade from A to C (*P* = 0.012), while three morphologic parameters (blastocoele expansion degree, inner cell mass (ICM) and TE) showed no statistical significance with miscarriage after multivariable analysis. The rate of aneuploidy was 47.7% (83 of 174) in total miscarried conceptuses. For euploid miscarriages, the grade B of ICM occupied a higher proportion compared with aneuploidy, with OR of 2.474, (95% CI, 1.311–4.699), *P* = 0.005.

**Conclusions:**

Chromosomal aberration of embryo is an important genetic factor for first trimester miscarriage, and the quality of ICM is a potential indicator for euploid miscarriage. Blastocysts with grade A of ICM should be given priority during single blastocyst transfer to reduce potential miscarriage.

## Introduction

The quality of embryos plays significant roles in establishing a viable pregnancy. High quality embryos have been validated to promote clinical pregnancy and live birth of infertile females in in vitro fertilization (IVF) cycles [1]. The development of assisted reproductive technology, including prolonged embryo culture and blastocyst vitrification, provided more optional methods to improve clinical outcomes [2, 3]. A well-established blastocyst culture system ensured the dependable single blastocyst stage transfer, which had been identified to be clinically superior to single cleavage stage embryo transfer [4, 5]. Moreover, single blastocyst transfer can significantly reduce the risk of twin or multiple pregnancy in assisted reproductive treatment [6].

Currently, viable blastocyst to be transferred is mainly evaluated through morphologic grading and morphokinetic development. According to the Gardner’s grading system [1], blastocyst morphology has been validated to predict clinical outcomes by previous studies. Top quality blastocyst transfer is associated with the higher implantation and ongoing pregnancy rate compared with non-top quality blastocyst [7]. Three morphologic parameters, the blastocoele expansion degree and the grade of ICM and TE, have been reported to predict clinical pregnancy and live birth for fresh and frozen blastocyst transfer cycles in various studies [8–11], which provide convenient guidance in the selection of viable embryos. Another important pregnancy outcome is miscarriage, as an unexpected condition, which even troubles many infertile couples lasting for years. However, influence factors of miscarriage based on blastocyst morphologic standard are still not fully understood in previous studies. Some studies proposed the morphology quality of ICM or TE is associated with pregnancy loss [7, 12–14], but others concluded there is no correlation between blastocyst morphology and miscarriage [15–17]. Therefore, the determination of blastocyst morphologic parameters related to miscarriage is helpful to improve the adverse pregnancy outcome of IVF patients.

Aneuploidy of embryos was identified to be an important factor of decreasing the implantation and increasing miscarriage rates. The correlation between chromosomal complement of preimplantation embryos and blastocyst morphology has been explored according to previous studies [18, 19], which provided clinical guidance for selection of blastocysts. However, the distribution of morphologic parameters among different chromosomal karyotypes of miscarried conceptuses has not yet been discussed. We expected to obtain clinical value by exploring the difference of blastocyst morphology corresponding to euploid and aneuploid miscarriage.

Our study focused on the association between blastocyst morphologic grading and miscarriage in single blastocyst transfer cycles during treatment with IVF or intracytoplasmic sperm injection (ICSI), and further investigated the distribution of morphologic parameters according to euploid and aneuploid of miscarried conceptuses. We validated chromosomal aberration of embryo is an important factor for miscarriage, and speculated the grade A of ICM should be given priority to reduce the risk of miscarriage in single blastocyst transfer.

## Materials and methods

### Study design

This study was a retrospective cohort analysis of 2873 intrauterine pregnancy cycles with single blastocyst transfer performed from January 2013 to April 2019 at the Reproductive Medical Center of the First Affiliated Hospital of Zhengzhou University in China. The data were from the Clinical Reproductive Medicine Management System/Electronic Medical Record Cohort Database (CCRM/EMRCD) in the Reproductive Medical Center. This retrospective analysis was approved by the Hospital Ethics Committee, and written informed consent was obtained from all patients before the beginning of clinical cycles. The exclusion criteria were [1] advanced age (females≥35 years old or males≥40 years old at transfer) [2]; cycles undergoing preimplantation genetic testing [3]; oocyte or sperm donation cycles [4]; embryo undergoing vitrification and warming before reaching the blastocyst stage [5]; abnormal chromosome karyotype for either of the couple [6]; uterine factors (malformation, endometriosis, adenomyosis or submucous myoma) [7]; recurrent pregnancy loss (defined as two or more consecutive spontaneous abortion with the same spouse) [8]; monozygotic twins or triplets pregnancy [9]; hypogonadotropic endocrine disfunction.

### ART procedure

Pituitary suppression and proper ovarian stimulation were performed with gonadotropin-releasing-hormone (GnRH) agonist and gonadotropin based on the previous protocol [9]. Trigger of promoting oocyte maturation was performed with the administration of human chorionic gonadotropin (hCG, Ovitrelle, Merck Serono) when two or more leading follicles were ≥ 18 mm in diameter, followed by transvaginal ultrasound-guided oocyte retrieval 36 to 38 h later. Fertilization was performed with conventional IVF or ICSI technology, and fertilized embryos were cultured in the G1 and G2 medium with an incubator under 5% O_2_, 6% CO_2_ and 89% N_2_ until day 5 or day 6. Fresh transfer was performed within 2 h of blastocyst grading and only with blastocyst of day 5. Vitrification was used for surplus blastocysts or cycles unsuitable for embryo transfer based on the endometrium thickness, hormone levels and ovarian stimulation of patients. Before vitrification, laser induced artificial shrinkage was performed [20]. The protocol of vitrification and warming followed the instructions established by Kuwayama et al. [21]. The survival of blastocyst was considered as blastocoele re-expansion over the half of total volume. For vitrified blastocysts, laser-assisted hatching was conducted to acquire a thin zona pellucida except for hatching and hatched blastocysts. Endometrial preparation and luteal supplement were performed with previous study [9].

### Blastocyst grading

Blastocysts were mainly evaluated based on three morphologic parameters: the blastocoele expansion degree, the ICM grade and the TE grade according to the Gardner and Schoolcraft’s grading system [1]. The degree of blastocoele expansion was categorized as following: 1, the early blastocyst with its blastocoele less than half of the total volume; 2, the early blastocyst with its blastocoele more than half of the total volume; 3, the blastocoele filled the entire blastocyst; 4, the expanded blastocyst with its thin zona pellucida; 5, the hatching blastocyst out of the zona pellucida; 6, the completely hatched blastocyst. The ICM grade followed: A, many tightly packed cells; B, several loosely packed cells; C, very few cells. The TE grade followed: A, many cells forming a dense epithelium; B, a few cells forming a loose epithelium; C, very few cells. The evaluation was performed by at least two professional embryologists.

### Clinical outcome and SNP analysis of chorionic villi

Serum hCG testing was performed on the day 14 and 18 after blastocyst transfer. Ultrasound monitoring was conducted to detect the gestational sac and fetal heart after 35 days of transfer. Clinical pregnancy was defined as the observation of a gestational sac inside the uterine cavity via ultrasound. First trimester miscarriage was defined as spontaneous pregnancy loss not more than 12 weeks of gestation after the observation of intrauterine gestational sac. 174 chorionic villi of miscarried conceptuses were performed SNP array analysis using a Human CytoSNP-12v2.1 Array (Illumina). Data analysis was carried out via Genome-Studio (Illumina 2011) and Karyo-Studio v1.4. Copy number variants (CNVs) of chorionic villi were mapped via DGV database to find abnormal CNVs. All steps were checked by at least two laboratorial technicians.

### Statistical analysis

Statistical analysis was performed using IBM SPSS Statistics 25. Continuous variables were presented as means ± standard deviation, with Student *t*-tests or Wilcoxon rank-sum test for differences between groups. Categorical data were presented as frequency and percentage, with chi-square and logistic regression analysis for differences between groups. All *P* values were two sided, and a *P* value of < 0.05 was considered as statistical significance.

## Results

### Patient and cycle characteristics for all study subjects

**Fig. 1:**
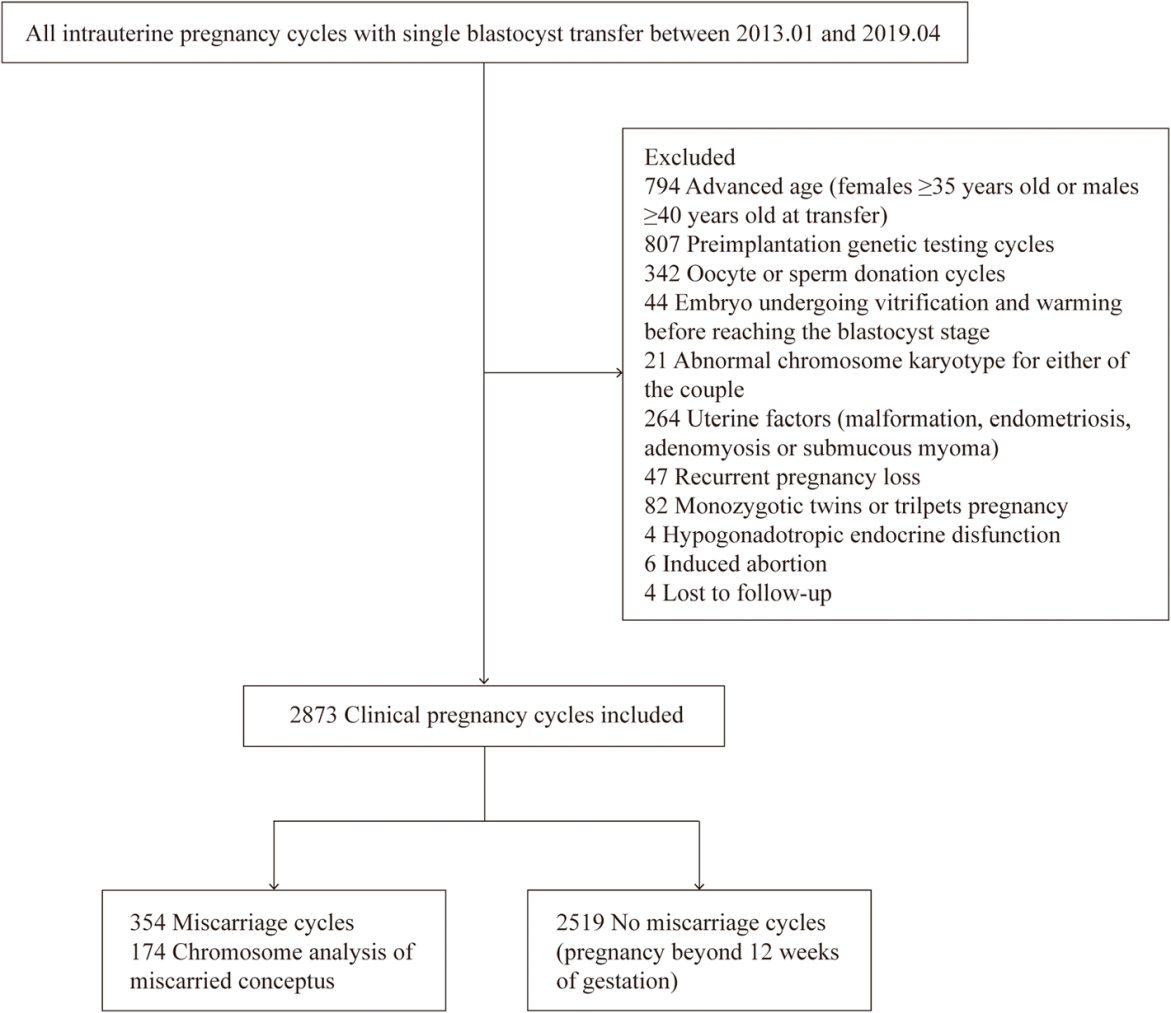
Inclusion and exclusion of study population

A total of 2873 clinical pregnancy cycles with single blastocyst transfer were included, grouped by 354 first trimester miscarriage cycles and 2519 cycles of pregnancy beyond 12 weeks of gestation as shown in Fig. 1. The characteristics of this study population and cycles were presented in Table 1 for miscarriage group and no miscarriage group. There was no statistical difference for two groups among prior gravidity and parity, body mass index (BMI), basal FSH, infertility diagnosis and fertilization method. Patients with advanced age were excluded in the study, but maternal and paternal ages of miscarriage group still presented statistical higher than no miscarriage group (28.6 ± 3.1 y versus 28.2 ± 3.0 y, *P* = 0.006 for maternal age, 30.0 ± 3.7 y versus 29.4 ± 3.5 y, *P* = 0.007 for paternal age). Endometrial thickness was thinner in the miscarriage group than no miscarriage group (11.2 ± 2.5 mm versus 11.7 ± 2.6 mm, *P* = 0.001). Frozen cycles presented higher miscarriage rate than fresh cycles (16.5% versus 9.3%, *P* < 0.001), and blastocysts of day 6 also showed higher miscarriage rate than day 5 (21.2% versus 11.3%, *P* < 0.001).

**Table 1:** Characteristics of patients and blastocyst morphology for all clinical pregnancy cycles

Variable	Miscarriage (***n*** = 354)	No miscarriage (***n*** = 2519)	***P*** value
Maternal age at oocyte retrieval, y	28.6 ± 3.1	28.2 ± 3.0	0.006
Prior gravidity	0.8 ± 1.0	0.8 ± 1.0	0.283
Prior parity	0.2 ± 0.4	0.2 ± 0.5	0.692
Body mass index (BMI), kg/m^2^	23.2 ± 3.4	23.0 ± 5.1	0.180
Basal FSH, mIU/mL	6.3 ± 1.8	6.2 ± 1.6	0.359
Paternal age, y	30.0 ± 3.7	29.4 ± 3.5	0.007
Endometrial thickness, mm	11.2 ± 2.5	11.7 ± 2.6	0.001
Infertility diagnosis, n (%)			0.310
Tubal factor	117 (33.1)	954 (37.9)	
Ovulatory disfunction	56 (15.8)	316 (12.5)	
Diminished ovarian reserve	8 (2.3)	51 (2.0)	
Male factor	81 (22.9)	547 (21.7)	
Unknown factor	92 (26.0)	651 (25.8)	
Fertilization method, n (%)			0.392
IVF	257 (72.6)	1773 (70.4)	
ICSI	97 (27.4)	746 (29.6)	
Cycle type, n (%)			< 0.001
Fresh	153 (43.2)	1499 (59.5)	
Frozen	201 (56.8)	1020 (40.5)	
Development days of blastocyst, n (%)			< 0.001
Day 5	292 (82.5)	2288 (90.8)	
Day 6	62 (17.5)	231 (9.2)	
Expansion degree, n (%)			0.496
1	3 (0.8)	26 (1.0)	
2	21 (5.9)	101 (4.0)	
3	92 (26.0)	699 (27.7)	
4	227 (64.1)	1586 (63.0)	
5	9 (2.5)	93 (3.7)	
6	2 (0.6)	14 (0.6)	
ICM grade, n (%)			0.404
A	124 (35.0)	940 (37.3)	
B	230 (65.0)	1579 (62.7)	
TE grade, n (%)			0.012
A	46 (13.0)	423 (16.8)	
B	201 (56.8)	1503 (59.7)	
C	107 (30.2)	593 (23.5)	

Values are presented as means ± SD or frequency (percentage)

### Blastocyst morphology and miscarriage

As shown in Table 1, the distribution of three blastocyst morphologic parameters was also described based on the grade of blastocoele expansion, ICM and TE for miscarriage group and no miscarriage group. Positive association was identified for miscarriage rate with decreasing TE grade (9.8% versus 11.8% versus 15.3%, *P* = 0.012). For univariable logistic analysis, the grade C of TE showed higher miscarriage risk than grade A, with OR of 1.659, (95% CI, 1.149–2.396), *P* = 0.007. After adjusted for maternal age at oocyte retrieval, paternal age, endometrial thickness, cycle type and development days, three morphologic parameters (expansion degree, ICM and TE) showed no statistical significance with miscarriage. The frozen cycle showed significant risk for miscarriage compared with fresh cycle, with OR of 1.605, (95% CI, 1.223–2.104), *P* = 0.001. The blastocyst of day 6 was identified to have significant risk for miscarriage compared with day 5 in this study, with OR of 1.519, (95% CI, 1.077–2.144), *P* = 0.017. All results of logistic analysis for factors related to miscarriage were shown in Table 2.

**Table 2:** Logistic analysis of factors related to miscarriage

Variable	Miscarriage rate, %(n)	Crude OR (95% CI)	***P*** value	Adjusted OR (95% CI)	***P*** value
Maternal age at oocyte retrieval, y		1.054 (1.016–1.093)	0.005	1.041 (0.991–1.093)	0.108
Paternal age, y		1.046 (1.014–1.079)	0.005	1.012 (0.970–1.056)	0.582
Endometrial thickness, mm		0.923 (0.882–0.965)	< 0.001	0.972 (0.924–1.021)	0.258
Cycle type					
Fresh	9.3 (153)	1		1	
Frozen	16.5 (201)	1.931 (1.542–2.417)	< 0.001	1.605 (1.223–2.104)	0.001
Development days of blastocyst					
Day 5	11.3 (292)	1		1	
Day 6	21.2 (62)	2.103 (1.549–2.855)	< 0.001	1.519 (1.077–2.144)	0.017
Expansion degree					
1 + 2	15.9 (24)	1.838 (0.861–3.925)	0.116	2.084 (0.956–4.541)	0.065
3	11.6 (92)	1.280 (0.663–2.471)	0.461	1.395 (0.712–2.735)	0.332
4	12.5 (227)	1.392 (0.737–2.630)	0.308	1.608 (0.839–3.082)	0.153
5 + 6	9.3 (11)	1		1	
ICM grade					
A	11.7 (124)	1		1	
B	12.7 (230)	1.104 (0.875–1.394)	0.404	0.859 (0.642–1.149)	0.306
TE grade					
A	9.8 (46)	1		1	
B	11.8 (201)	1.230 (0.877–1.724)	0.230	1.207 (0.827–1.762)	0.328
C	15.3 (107)	1.659 (1.149–2.396)	0.007	1.509 (0.953–2.389)	0.079

### Distribution of morphologic parameters among euploid and aneuploid miscarried conceptuses

The chromosomal SNP analysis was performed for a total of 174 miscarried chorionic villi in the preimplantation genetic diagnosis center. The chromosomal aberrance rate was 47.7%. There was no statistical difference for euploid and aneuploid conceptuses among maternal and paternal ages, BMI, infertility diagnosis, fertilization method, cycle type and development days. According to the chi-square analysis, the chromosome karyotype of miscarried conceptus and the ICM grade of transferred blastocyst were significantly correlated (*P* = .005) (Table 3). Miscarried conceptus with normal chromosomal karyotype showed more proportion of the ICM grade B compared with abnormal chromosomal karyotype, with OR of 2.474, (95% CI, 1.311–4.699). No statistical correlation was found between the chromosome karyotype and the expansion degree and TE grade, but the grade C of TE presented higher proportion in euploid miscarriage than in aneuploidy (30.8% versus 21.7%).

**Table 3:** Characteristics of patients and blastocyst morphology grouped by chromosome karyotype of miscarried conceptus

Variable	Euploidy (***n*** = 91)	Aneuploidy (***n*** = 83)	***P*** value
Maternal age at oocyte retrieval, y	28.2 ± 3.2	29.0 ± 3.1	0.088
Body mass index (BMI), kg/m^2^	23.4 ± 2.9	22.5 ± 3.3	0.065
Paternal age, y	29.4 ± 3.5	30.2 ± 3.6	0.142
Infertility diagnosis, n (%)			0.611
Tubal factor	23 (25.3)	23 (27.7)	
Ovulatory disfunction	22 (24.2)	13 (15.7)	
Diminished ovarian reserve	1 (1.1)	2 (2.4)	
Male factor	22 (24.2)	19 (22.9)	
Unknown factor	23 (25.3)	26 (31.3)	
Fertilization method, n (%)			0.207
IVF	60 (65.9)	62 (74.7)	
ICSI	31 (34.1)	21 (25.3)	
Cycle type, n (%)			0.074
Fresh	36 (39.6)	45 (54.2)	
Frozen	55 (60.4)	38 (45.8)	
Development days, n (%)			0.575
Day 5	75 (82.4)	71 (85.5)	
Day 6	16 (17.6)	12 (14.5)	
Expansion degree, n (%)			0.709
1	1 (1.1)	0	
2	2 (2.2)	1 (1.2)	
3	21 (23.1)	23 (27.7)	
4	62 (68.1)	57 (68.7)	
5	4 (4.4)	2 (2.4)	
6	1 (1.1)	0	
ICM grade ^a^, n (%)			0.005
A	24 (26.4)	39 (47.0)	
B	67 (73.6)	44 (53.0)	
TE grade, n (%)			0.346
A	14 (15.4)	12 (14.5)	
B	49 (53.8)	53 (63.9)	
C	28 (30.8)	18 (21.7)	

Values are presented as means ± SD or frequency (percentage)

^a^ Univariable logistic analysis for ICM grade B: euploid miscarriage versus aneuploid miscarriage, OR (95% CI), 2.474 (1.311–4.669), *P* = 0.005

## Discussion

Assisted reproductive technology (ART) is determined to promote live birth rate of infertile couples and reduce the pregnancy loss. Blastocyst morphology was identified to be correlated to implantation, pregnancy and live birth for fresh and frozen blastocyst transfer cycles [8–11]. However, the mechanism underlying the grade of morphology parameters and miscarriage is still not fully understood. In a frozen-thawed single blastocyst transfer with first IVF treatment, the TE morphology was significantly to be correlated to the rates of ongoing pregnancy and miscarriage [13], but the stage of vitrification for partial embryos was pronuclear stage, which may decreased the reliability of the conclusion as a confounding factor. An earlier retrospective cohort study identified transferred blastocysts with poor ICM and fragmentation showed a higher miscarriage rate [12]. And based on a secondary analysis of data prospectively collected in a large multicenter trial, the ICM grade was significantly associated with early pregnancy loss [14]. Another study including euploid blastocyst transfer also identified the grade C of ICM yielded a statistically significantly higher miscarriage rate than the grade A or B [7]. However, the limited sample size precluded definite conclusion. Moreover, no correlation was found between morphology grade and miscarriage based on several recent studies in fresh single blastocyst transfer cycles, frozen cycles or preimplantation genetic screening cycles [15–17]. The inconsistence of these conclusions needs further analysis with fewer confounding factors to elucidate the effect of blastocyst morphology on miscarriage.

Our study identified there was no statistical correlation between miscarriage and three blastocyst morphologic parameters, including the degree of blastocoele expansion and the grade of ICM and TE, after multivariable logistic analysis. Combined previous studies focusing on the implantation rate of blastocyst transfer, we speculated the main effect of morphology parameters on the clinical outcome was the blastocyst implantation rather than clinical pregnancy loss. High quality blastocyst ensured the viable implantation and development, while the pregnancy loss maybe caused by other potential factors. The miscarriage rate of the fresh cycles was significantly lower than the rate of the frozen cycles in this study (9.3% versus 16.5%, *P* = 0.001). Previous studies also showed that the early miscarriage rate increased in frozen embryo transfer cycles compared with fresh cycles in the patients younger than 35 years old [22, 23]. That may be explained by the down-regulation of GnRH agonist that provide it a rest to restore the uterine capacity for embryo implantation, especially for younger women [24]. Another experiment on mice indicated that ovarian stimulation with GnRH agonist could partially restore the endometrial secretion and improve uterine receptivity [25]. Therefore, the extra-pituitary GnRH agonist on the uterine environment provided better receptivity for endometrium in the fresh cycles. In addition, the fresh embryos transfer was prior for the patients with ART treatment in our clinical center, while the patients with surplus vitrified embryos or previous failure cycles would be performed with the frozen transfer. Therefore, the quality of blastocysts performed with fresh transfer was relatively better than that performed with frozen transfer, which might also be the most important factor for poor grade of TE associated with miscarriage rate via univariable analysis. The selection of blastocyst transfer was only day 5 for fresh cycles in our clinical center, and meanwhile day 5 and day 6 for frozen cycles, thus present study also showed a statistical higher miscarriage rate in blastocysts of day 6 than day 5 (*P* = 0.017).

Aneuploidy has been validated as one of the most important factors for embryo development and miscarriage. We analyzed the chromosome karyotypes of 174 miscarried conceptuses based on SNP array analysis, the total chromosome aberrance rate was 47.7%, proving the importance of euploidy for viable pregnancy. In addition, miscarried conceptus with normal chromosomal karyotype showed more proportion of the ICM grade B compared with abnormal chromosomal karyotype, reflecting the important effect of high grade ICM on embryo development and maintaining gestation. Poor vitality of the ICM potentially carried the risk of pregnancy loss, which was consistent with the idea of Irani et al. [7], poor grade of the ICM yielded a statistically significantly higher miscarriage rate than good or average grade of the ICM for the euploid blastocyst transfer. Our study elucidated the statistical difference of the ICM on euploid miscarried conceptuses compared to aneuploid ones, and speculated the quality of ICM might be one factor for arrested pregnancy of euploid fetuses without the beat of fetal heart. The discordance of ICM quality in euploid and aneuploid miscarriage probably puzzled the impact of morphologic parameters on total miscarriage rate. Previous studies also identified aneuploid human blastocysts showed a higher percentage with poor quality ICM, TE and expansion grades, compared to euploid blastocysts [19, 26]. It is fairly well known that the blastocysts with both poor grade of morphology and abnormal chromosome would be partially weeded out during the implantation of embryos. After the clinical pregnancy, embryos with either abnormal karyotypes or poor vitality of the cells still produced the possibility of miscarriage. Therefore, euploid embryos occupied important roles in the establishment of reliable pregnancy, the grade of the ICM potentially associated with the maintenance of pregnancy according to the more grade B of ICM than grade A in euploid miscarried conceptuses.

Advantages of our analysis included the strict inclusion and exclusion criteria for statistical study. Potential interference factors, such as advanced age, endometriosis and adenomyosis, history of recurrent pregnancy loss, genetic factors and uterine malformation, were total excluded for precise study of the association between the blastocyst morphology and miscarriage and karyotype of miscarried conceptus in single blastocyst transfer cycles. The large sample size provided a better convincing conclusion about the impact of morphologic parameters on the miscarriage. Combined the chromosomal karyotype analysis of miscarried conceptuses, this study firstly explored the correlation between blastocyst morphology and karyotype of miscarried tissues, discussed different morphologic distribution of ICM in euploid and aneuploid miscarriage, and provided a potential guidance for single blastocyst transfer from the point of karyotype of miscarried conceptuses. The guidance for the selection of blastocysts and avoidance of pregnancy loss should be evaluated in the further studies.

Limitations are inevitable in this retrospective study. Assisted hatching was identified to have good evidence of slightly improving the clinical pregnancy rates, especially in poor prognosis patients, but have insufficient evidence of improving live birth rates [27]. Blastocysts performed assisted hatching were also included in our analysis, and in previous studies no sufficient evidence showed assisted hatching would influence the pregnancy loss. In addition, not all patents were treated for the first ART cycle, and 73 patients of single blastocyst transfer were performed two or more cycles in our analysis, the difference of individual characteristics might influence the precise statistical result. The CC grade of blastocyst morphology cannot be transferred for patients in our reproductive clinic. Therefore, the quality of blastocysts for transferring was relative good or average quality, a lack of poor quality of the ICM and TE for analysis in this study. Further studies must be performed in large prospective randomized controlled trials.

## Conclusion

In conclusion, the quality of ICM is a potential factor for euploid miscarriage, and blastocysts with grade A of ICM should be given priority during the IVF/ICSI cycles to reduce the miscarriage. Chromosomal aberration of embryo is an important factor for miscarriage. For embryos with normal chromosome, the quality of the ICM might provide more value in miscarriage in single blastocyst transfer cycles.

## Data Availability

The data used during this study are available from the corresponding author on reasonable request.

## Abbreviations

ART: Assisted reproductive technology
BMI: Body mass index
CNVs: Copy number variants
GnRH: Gonadotropin-releasing-hormone
hCG: Human chorionic gonadotropin
ICM: Inner cell mass
ICSI: Intracytoplasmic sperm injection
IVF: In vitro fertilization
TE: Trophectoderm
